# A new insight: crosstalk between neutrophil extracellular traps and the gut-liver axis for nonalcoholic fatty liver disease

**DOI:** 10.3389/fimmu.2025.1599956

**Published:** 2025-06-27

**Authors:** Jinping Yin, Yanli Zhu, Rongrong Liu, Weixin Wang, Zhicheng Wang, Jianfeng Wang

**Affiliations:** ^1^ Department of Radiotherapy in China-Japan Union Hospital of Jilin University, Changchun, Jilin, China; ^2^ National Health Commission (NHC) Key Laboratory of Radiobiology, School of Public Health, Jilin University, Changchun, Jilin, China

**Keywords:** inflammation, liver disease, gut microbiota, pathophysiological progression, immune dysregulation

## Abstract

Non-alcoholic fatty liver disease (NAFLD) is a widespread chronic liver disorder, affecting nearly a quarter of the global population. It progresses from simple steatosis to non-alcoholic steatohepatitis (NASH), fibrosis, cirrhosis, and hepatocellular carcinoma (HCC). The gut-liver axis is crucial in NAFLD progression, driven by intestinal barrier dysfunction, microbial translocation, and immune dysregulation. Neutrophil extracellular traps (NETs)—web-like structures of DNA, histones, and inflammatory proteins—promote chronic inflammation and liver injury. This review examines the role of NETs in gut-liver axis crosstalk and NAFLD progression. It explores how NETs amplify inflammation, contribute to fibrosis, and facilitate the progression from NAFLD to HCC by interacting with gut microbiota and immune signaling pathways. Therapeutic strategies targeting NETs, such as reducing their formation, enhancing degradation, and modulating the gut microbiota, offer promising approaches to mitigate disease progression. This review sheds light on the interplay between NETs and the gut-liver axis, offering new insights into NAFLD pathophysiology and potential therapeutic strategies to improve patient outcomes.

## 1 Introduction

Non-alcoholic fatty liver disease (NAFLD) is now one of the most common chronic liver diseases, affecting one billion people worldwide (1). It ranges from non-alcoholic fatty liver (NAFL) to the more severe non-alcoholic steatohepatitis (NASH), which can progress to fibrosis, cirrhosis, and hepatocellular carcinoma (HCC) (2). Intestinal barrier dysfunction and bacterial translocation through the gut-liver axis drive liver disease progression (3). This bidirectional network connects the liver and intestine via the biliary tract, portal circulation, and systemic circulation, interacting closely with the immune system and gut microbiota (4, 5). Its dysfunction disrupts immune balance, worsening liver disease (6, 7).

Over the past two decades, the role of immune cells in NAFLD progression and fibrosis has gained increasing attention (8, 9). Early studies identified inflammation as a key driver of NAFLD leading to extensive research on the contributions of immune cell types and inflammatory factors (10–16).

Neutrophils, the most abundant immune cells, constitute about 70% of circulating leukocytes and serve as the first line of defense in innate immunity (17). They rapidly migrate to sites of infection or injury, but their persistent activation and excessive recruitment contribute to various inflammatory diseases (18). Neutrophils promote tissue damage by releasing proteases such as matrix metalloproteinases and neutrophil elastase and generating oxidative bursts that disrupt cell membranes (19). They also form neutrophil extracellular traps (NETs), web-like structures of DNA, histones, and inflammatory proteins that drive chronic inflammation and cancer progression (20, 21). Emerging evidence links NETs to the gut-liver axis, highlighting their role in sustaining inflammation and promoting the progression of NAFLD to advanced liver disease, including HCC (22, 23).

This review examines the role of NETs in NAFLD pathophysiology, focusing on their interactions with the gut-liver axis and how these contribute to disease onset and progression. In addition, we will explore NETs’ involvement in the transition from normal liver to NAFLD, NASH, liver fibrosis, and ultimately HCC. Additionally, it discusses potential therapeutic strategies targeting NETs and intestine, including inhibiting their formation, promoting degradation, modulating the gut microbiota, and employing multi-targeted combination therapies. By elucidating the crosstalk between NETs and the gut-liver axis, this review aims to uncover novel pathophysiological mechanisms and therapeutic opportunities for NAFLD and its complications.

## 2 NET formation and functional roles

### 2.1 Formation of NETs

NETs are highly negatively charged, web-like structures released by activated neutrophils (24). The process of their formation, termed NETosis, has been the subject of extensive research in recent years. It is now understood that NETosis occurs through three distinct mechanisms (**Table 1**).

**Table 1:** Three mechanisms for the formation of NETs.

Type	Occurrence	Stimuli	Main processes	DNA for the formation of NETs	Dependency	Accompanied by cell death or not
Suicidal NETosis	2–4 h after activation	PMA, ionomycin, crystals, CXCL8, Fc-receptors, pathogens	1. Activates protein kinase C (PKC) and the Raf-MEK-ERK-MAP kinase pathway, generating ROS to transfer NE and MPO to the nucleus; 2. Ca^2+^ activates PAD4 which induces chromatin decondensation	Nuclear DNA	NOX-dependent	Yes
Vital NETosis	5–60 min after activation	S. aureus, LPS, GM-CSF and C5a, conditioned media of thyroid cancer, immune complexes	Nuclear DNA fuses with cytoplasm followed by the budding of vesicles	Nuclear DNA	NOX-independent	No
Mitochondrial NETosis	15 min after activation	GM-CSF, LPS,C5a	Mitochondrial DNA is released to form NETs	Mitochondrial DNA	Mitochondria-dependent	No

Suicidal NETosis, the first type of NETosis, is NADPH oxidase-dependent and results in neutrophil death, typically occurring within 2–4 hours of neutrophil activation. Stimulation by Phorbol Myristate Acetate (PMA) (25), ionomycin (26), crystals (27), CXCL8 (28), Fc receptors (29, 30) and pathogens activates the NADPH oxidase complex via the PKC-Raf-MERK-ERK pathway (31). This activation generates reactive oxygen species (ROS), which promote chromatin decondensation and facilitate the transfer of neutrophil elastase (NE) and myeloperoxidase (MPO) to the nucleus (32). Additionally, extracellular Ca^2+^ influx activates peptidylarginine deaminase 4 (PAD4), leading to histone citrullination, which weakens the electrostatic bond between histones and DNA, further promoting chromatin decondensation (26, 33). Ultimately, the nuclear membrane ruptures, expelling nuclear contents that fuse with cytoplasmic granules to form NETs.

The second type of NETosis, termed vital NETosis, occurs independently of NADPH oxidase and does not involve neutrophil death. It is typically induced within 5–60 minutes following neutrophil activation. This process is triggered by Toll-like receptor 2 (TLR2), Toll-like receptor 4 (TLR4), and the complement protein C3 in response to S. aureus (34, 35), as well as by lipopolysaccharides (LPS) (32, 36–40), granulocyte/macrophage colony-stimulating factor (GM-CSF) and complement factor 5a (C5a) (41, 42), conditioned media from thyroid cancer cells (43), and immune complexes (42, 44, 45). During vital NETosis, nuclear DNA merges with the cytoplasm and is subsequently released via vesicle budding. Despite nuclear extrusion, these neutrophils retain their phagocytic capacity, and their lifespan remains unaffected by DNA loss (46).

The third type, mitochondrial NETosis, typically occurs within 15 minutes of neutrophil activation. Upon stimulation by GM-CSF, LPS, or C5a, neutrophils release mitochondrial, rather than nuclear, DNA to form NETs without undergoing cell death (41).

### 2.2 Structure of NETs and its role in NAFLD progression

Upon activation, neutrophils undergo morphological changes, becoming flattened as nuclear lobules disappear, chromatin decondenses, and the inner and outer nuclear membranes separate. The nuclear membrane then fragments into vesicles, while the nucleoplasm and cytoplasm merge into homogeneous clumps. Eventually, the cell condenses, becoming round, and the cytoplasmic membrane ruptures, releasing intracellular components to form fibrous bundles (47). NETs display a unique ultrastructure, consisting of a chromatin filament framework, 15–17 nm in diameter, primarily composed of modified nucleosomes (24, 48). This filamentous network is interspersed with 50 nm globular structures. These globules are enriched with proteins from primary and secondary granules, including NE, MPO, cathepsin G, proteinase 3, BPI (cationic bactericidal/permeability-increasing protein), calgranulin, α-defensins, lactoferrin, LL-37 (a fragment of cathelicidin hCAP18), and PTX3. Additionally, tertiary granule components, such as matrix metalloproteinase-9 (MMP-9) and peptidoglycan recognition protein-S (PGRP-S), are incorporated (49–51).

**Figure 1:**
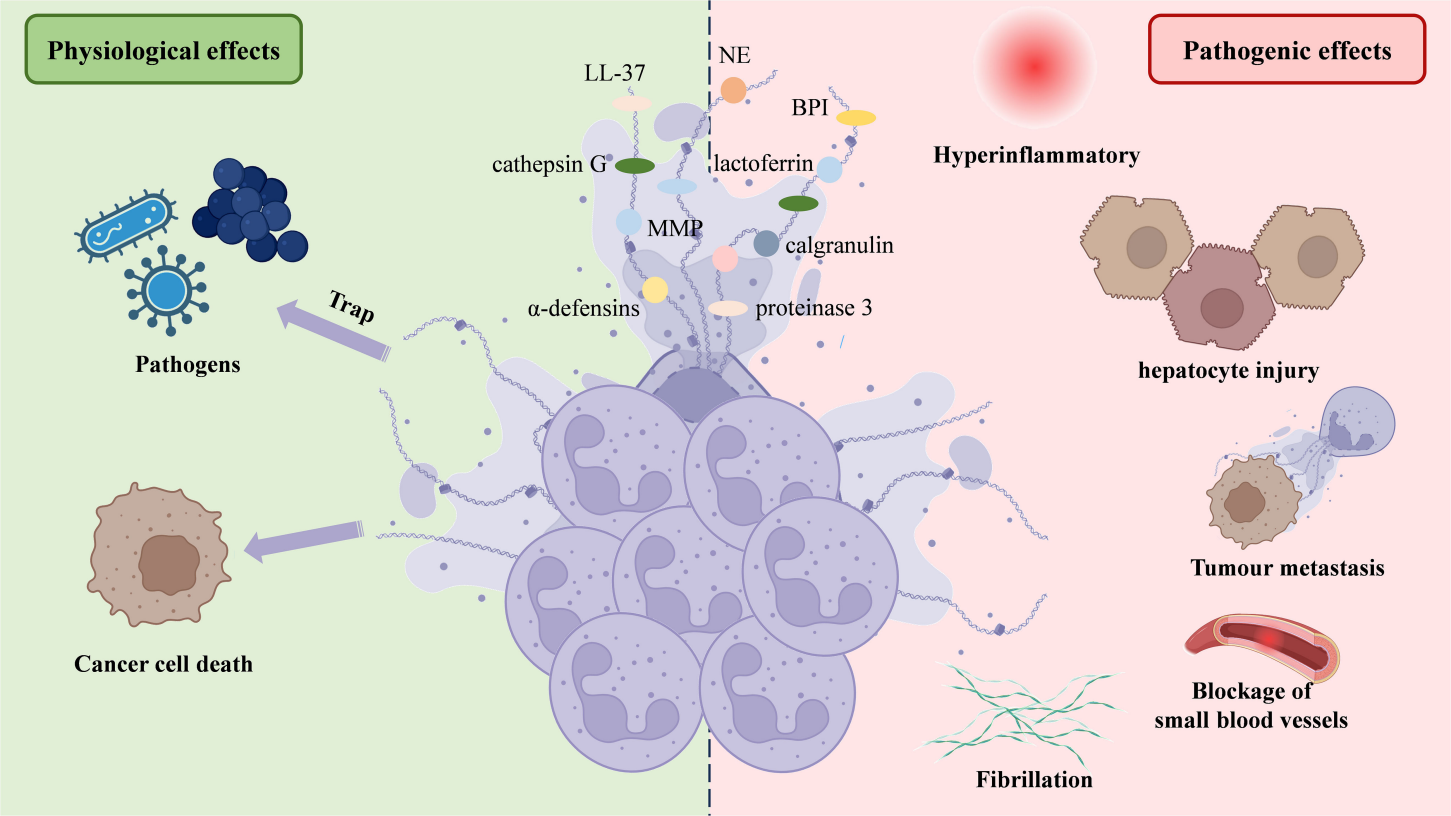
NETs’ structure and two-sided functionality. NETs are web-like structures formed by decondensed chromatin (DNA and histones) decorated with neutrophil-derived antimicrobial proteins and enzymes, such as NE, lactoferrin, α-defensins, and proteinase 3. NETs play a dual role in physiological and pathological processes. On the physiological side, NETs trap and kill pathogens, contributing to the immune defense, and induce cancer cell death. However, on the pathological side, excessive or dysregulated NETs can cause hyperinflammation, hepatocyte injury, promote tumor metastasis, lead to fibrillation, and obstruct small blood vessels, contributing to thrombotic and inflammatory diseases. NETs, Neutrophil extracellular traps; NE, Neutrophil elastase.

The role of NETs in NAFLD progression can be categorized into two main effects: physiological and pathogenic (**Figure 1**). Regarding physiological effects, both *in vivo* and *in vitro* studies have demonstrated that DNA fibrils from NETs can adhere to gram-negative and gram-positive bacteria, as well as fungi, significantly limiting pathogen transmission (24, 34, 36, 52). However, the ability of NETs to kill pathogens remains debated. While NETs contain bactericidal proteins and enzymes, such as BPI, LL-37, α-defensins, NE, MPO, protease 3, and cathepsin G, some studies have shown pathogen death within NETs (34, 53, 54). Others have not observed such effects, suggesting that plasma protease inhibitors, which inhibit enzymes like NE, and the presence of apolipoproteins, which impair the bactericidal activity of LL-37 and α-defensins, may limit their function (55, 56) (**Figure 1**).

In terms of pathogenic effects, excessive NET formation has been linked to increased liver inflammation, exacerbating NAFLD progression (57–59). Neutrophils can also mediate hepatocyte injury via NETs, ROS and inflammatory mediators (60). Primary tumors trigger neutrophil recruitment and NET release at pre-metastatic sites, enhancing tumor growth and spread by interacting with cytokines. This mechanism may explain liver colonization by colorectal, lung, and breast cancers (61). NET formation by vital NETosis boosts HMGB1 production in tumor cells, activating TLR9-dependent pathways (62) and the TLR4/9-COX2 pathway (63), which improve tumor cell survival and invasion. Some studies have demonstrated that tumor cells expressing advanced glycation end-product receptors bind HMGB1, activating nuclear factor kappa-B (NF-κB) signaling and inducing interleukin-8 (IL-8) release. This attracts additional neutrophils and promotes NET formation, thereby facilitating the hepatic spread of colorectal cancer (64, 65). Other research indicates that NETs contribute to thrombosis in HCC patients and may worsen liver surgery-induced distal organ damage by triggering a systemic procoagulant state and microvascular immune thrombosis (66), as confirmed in a mouse model of hepatic ischemia-reperfusion injury (IRI) (67). Zermatten et al. observed elevated NET levels in the plasma of cirrhotic patients, potentially due to impaired hepatic clearance (68). Zhao et al. found that S1PR knockdown alleviates liver inflammation and fibrosis by inhibiting NET formation (69). These findings highlight the significant role of NETs in promoting the development of NAFLD (**Figure 1**).

## 3 The gut-liver axis and NAFLD

The intestinal barrier is a crucial anatomical and functional structure that mediates interactions between the gut and liver. It restricts the spread of microbes and toxins while permitting the absorption of nutrients into circulation for delivery to the liver. The “multiple hits” hypothesis explains the pathogenesis of NAFLD, involving factors such as genetic predisposition, altered gastrointestinal hormone and adipokine secretion, insulin resistance, nutritional imbalances, gut microbiota dysbiosis, and inflammation (7, 70–72). Among these, NAFLD is particularly associated with increased intestinal permeability and shifts in gut microbiota, which further exacerbate disease progression (73). Intestinal vascular barrier dysfunction has been identified as a key factor in NAFLD development (74, 75). The mechanisms regulating gut-liver axis homeostasis are complex, involving dietary, genetic, and microbiota-related factors that collectively influence intestinal permeability and metabolite levels. The imbalance of gut-liver axis homeostasis leads to intestinal ecological disorders, and the close connection between intestinal epithelial cells is destroyed under the action of various adverse factors, permeability changes or exposure to toxic bacterial metabolites, and then the intestinal epithelium is damaged (76). Fatty liver disease, including NAFLD, NASH and the later stages of development, the pathogenesis of multiple factors interwoven into a network of different stages, different levels of interaction.

### 3.1 Primary or secondary changes in gut microbiota

Gut microbiota plays a critical role in maintaining gut-liver axis homeostasis. Disruptions in its composition and transport to the liver through the gut-liver axis contribute significantly to the development of NAFLD. The intestinal microbiota is predominantly composed of bacterial phyla, with Bacteroidetes and Firmicutes being the most abundant, as identified through advanced techniques like shotgun sequencing and ribosomal multi-site sequencing (77, 78). Studies have shown that gut microbiota composition is altered in NAFLD patients, with reduced relative proportions of Alistipes, Odoribacter, Rikenellaceae, Bacteroides, Oscillibacter, and lactic acid bacteria from Firmicutes, alongside an increase in Peptiophilus, Escherichia, Enterobacterium, and anaerobic bacteria (79).

These microbiota changes are influenced by factors such as mode of delivery, diet, lifestyle, drug use, and host genetics. Gut microbiota plays an essential role in immunity, digestion, endocrine functions, neurotransmission, drug metabolism, and endotoxin clearance. In NAFLD, alterations in gut microbiota are primarily driven by genetic and metabolic abnormalities (80). These disruptions exacerbate NAFLD progression by increasing intestinal permeability, releasing bacterial toxins, and causing metabolic disorders. Additionally, altered gut microbiota can reach the liver via the gut-liver axis, creating an inflammatory environment that promotes hepatic steatosis (6). Some studies have targeted the detrimental effects of gut dysbiosis in NAFLD by using microbiota-regulating drugs, such as glucagon-like peptide-1 receptor agonists (GLP-1 RAs) for type 2 diabetes (81). These treatments have shown effectiveness in reversing hepatocyte autophagy and reducing NAFLD-associated dysbiosis, further supporting the need to explore the role of gut microbiota in NAFLD (79).

There is bidirectional crosstalk between the gut microbiota and the liver through the gut-liver axis. The liver influences the gut microbiota by releasing hormones, bile, and antibodies into the intestine (7). In NAFLD, the accumulation of fat in the liver leads to lipotoxicity, disrupting metabolic processes. The regulating effect of substances secreted by the diseased liver on intestinal microbes causes changes in intestinal microbial structure, which can act on the liver again through various ways as secondary factors, causing further development or remission of NAFLD.

### 3.2 Increased intestinal permeability

Impairment of the intestinal barrier is a key factor in disrupting the gut-liver axis. The intestinal barrier comprises several components: the mechanical, biochemical, microbial, and immune barriers, which include intestinal epithelial cells, secretions, gut microbiota, gut-associated lymphoid tissue (GALT), and diffuse immune cells (82). The permeability of the intestinal barrier is influenced by various factors, such as the protective mucosal layer produced by goblet cells, antimicrobial peptides from Paneth cells, tight junction proteins maintaining epithelial integrity, and immune cell activation. The tight junctions, located at the apical end of epithelial cells, are critical for regulating intestinal mucosal permeability. Under pathological conditions, disruption of tight junction structure and function leads to impaired intestinal barrier function (83). Environmental factors, such as pollution (84), and dietary changes can exacerbate NAFLD by compromising the barrier, which allows the inappropriate transport of nutrients, bacteria, and toxins to the liver (85). In NAFLD patients, dysregulation of the microbiota results in a thinner mucosal layer, decreased antimicrobial peptide production, reduced tight junction protein levels, and changes in immune cell populations in the lamina propria (86). Macrophage activation triggers pro-inflammatory cytokine production and amplifies neutrophil responses, further increasing intestinal permeability and disrupting entero-hepatic axis homeostasis.

### 3.3 Increased metabolic endotoxin

Increased intestinal barrier permeability can induce metabolic endotoxemia, which in turn contributes to the development of steatohepatitis. Additionally, altered absorption of various metabolites can affect liver metabolism, promoting liver steatosis and fibrosis. In NAFLD patients, dysregulation of gut microbiota and heightened intestinal permeability result in greater liver exposure to bacterial products, thereby triggering metabolic endotoxemia and disrupting gut-liver axis function (87). A dysfunctional gut microbiota with increased intestinal permeability exposes the liver to bacterial compounds, including ethanol, short-chain fatty acids (SCFAs), pathogen-associated molecular patterns (PAMPs), and damage-associated molecular patterns (DAMPs) (88). These compounds, particularly DAMPs released from the compromised intestine, activate neutrophils and induce the formation of NETs through synergistic mechanisms (89–91).

### 3.4 Vicious cycle of NETs in intestinal diseases

The pathogenesis of various intestinal diseases involves multiple factors that promote excessive production of pro-inflammatory substances and immune responses, leading to pathological changes in the intestinal wall. Colorectal biopsies from patients with Crohn’s disease and ulcerative colitis revealed increased expression of NETs-associated proteins, such as PAD4, compared to healthy controls (92). Furthermore, treatment with infliximab, a high-affinity monoclonal antibody targeting tumor necrosis factor-α (TNF-α), resulted in elevated expression of these proteins (93). Studies demonstrated that inhibition of TNF-α reduced both NETs levels and PAD4 expression in patients with intestinal diseases, indicating elevated NETs in these conditions (93). Additionally, research using a mouse model showed that treatment with deoxyribonuclease I (DNase I) reduced NET release and alleviated colitis, as well as the development of colitis-related tumors (94). These findings suggest that NETs play a catalytic role in intestinal disease progression, forming a vicious cycle of mutual causality.

## 4 NETs-gut-liver axis interaction

**Figure 2:**
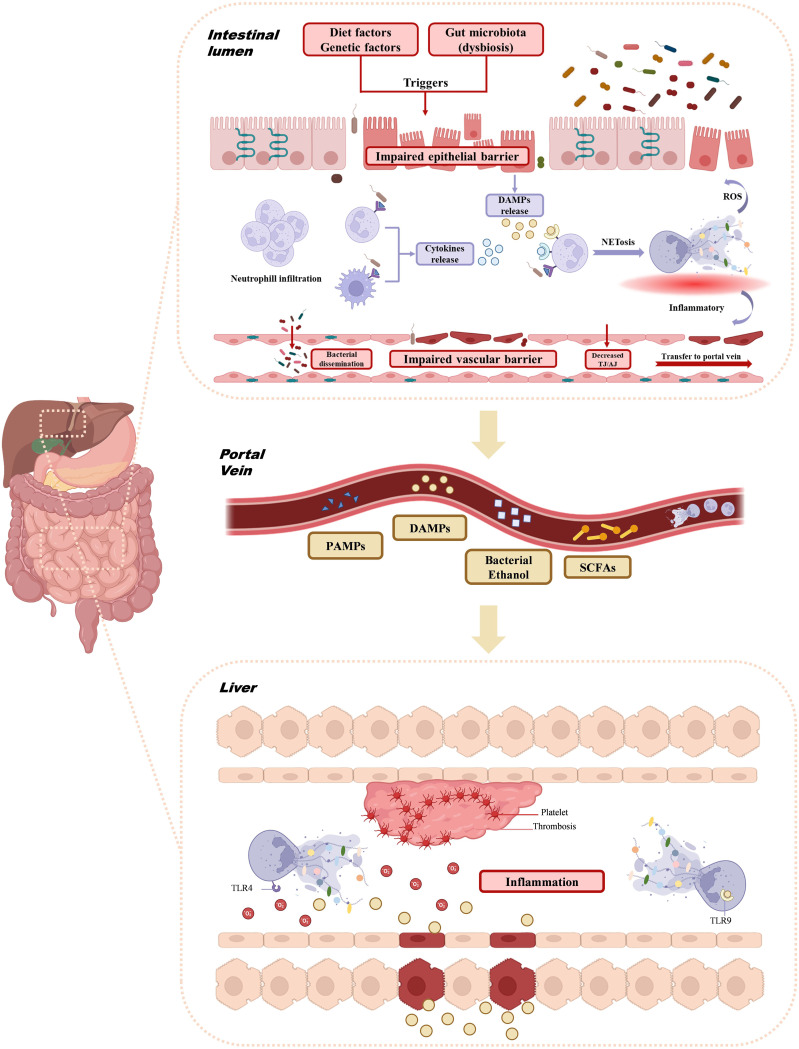
Interaction of NETs with gut-liver axis. Triggers such as diet, genetic factors, and gut microbiota dysbiosis impair the intestinal epithelial barrier, allowing neutrophil infiltration and promoting the formation of NETs. The resulting ROS and inflammatory environment further disrupt the intestinal barrier and portal vein. Concurrently, bacterial dysbiosis and barrier dysfunction enable bacterial products like PAMPs, DAMPs, ethanol, and SCFAs to enter the portal vein. These signals activate liver inflammation, trigger platelet aggregation via TLR4 and TLR9, and cause thrombosis and liver injury. NETs, Neutrophil extracellular traps; ROS, Reactive Oxygen Species; PAMPs, Pathogenetic associated molecular patterns; DAMPs, Damage-associated molecular patterns; SCFAs, Short-chain fatty acids; TLR, Toll-like receptor.

The gut and liver engage in a dynamic relationship, influenced by both organs, the microbiome, diet, and environmental factors. This interconnection is referred to as the gut-liver axis (95). The liver receives blood from the intestine through the portal vein, which supplies most of the venous and arterial blood to the liver. From a pathophysiological perspective, the intestinal mucosal barrier and the portal vein regulate the exchange of toxins and microorganisms between the gut and liver, allowing nutrients to enter circulation and reach the liver (7, 96). This portal vein-mediated interaction facilitates the transfer of bacterial metabolites from the intestine to the liver, potentially contributing to various liver diseases (95). Numerous studies have highlighted the gut-liver axis’ pivotal role in NAFLD progression (7). NASH patients, in particular, exhibit higher levels of intestinal microbiota imbalance, along with intestinal inflammation and barrier damage. Disruption of the mucosal transport and permeability, particularly at the tight junctions between intestinal endothelial cells (e.g., occludin and claudin), is observed (97). Dysfunction of the gut-vascular barrier is considered a key factor in NAFLD development (98, 99). Several studies indicate that gut dysbiosis can impair the intestinal epithelium, weaken tight junctions, increase intestinal permeability, and expose the liver to harmful bacterial metabolites (90, 100, 101). Both NAFLD and NASH are associated with increased intestinal barrier permeability and the translocation of bacteria or their products into the bloodstream (102–104). Given the crucial role of NETs in both processes, further investigation into their involvement in the gut-liver axis and NAFLD is warranted (**Figure 2**).

NETs are recognized as a key mechanism through which the gut-liver axis influences the progression of liver diseases, including NAFLD. The impairment of the intestinal epithelial barrier, triggered by factors such as diet, genetics, and gut microbiota dysbiosis, leads to the release of cytokines from various immune cells in an inflammatory environment (105). The dysregulated gut microbiota acts as a major trigger for the overactivation of neutrophils in the intestinal wall, resulting in NET formation. Recent studies have shown that microorganisms such as adherent-invasive Escherichia coli (AIEC) and Entamoeba histolytica can stimulate NETs (106, 107). Mouries et al. observed an initial disruption of the intestinal epithelial and gut vascular barriers (GVB) in NASH (3). During diet-induced dysbiosis, the gut vascular barrier becomes compromised (3). Gao et al. demonstrated that neutrophils infiltrate and release NETs in the gut of LPS-induced endotoxemic rats, and that DNase I administration, which disrupts NETs, alleviated intestinal epithelial cell apoptosis, intestinal damage, and the systemic inflammatory response (108). The formation and clearance of NETs is a dynamic process, and if this balance is disrupted, excessive NETosis can contribute to chronic inflammation (109), further damaging the intestinal barrier. NETs generate high levels of reactive oxygen species (ROS), leading to epithelial damage, activating redox-sensitive inflammatory pathways, and promoting bacterial translocation, which can damage the vascular barrier and lead to the release of TH/AJ (110). These factors contribute to the transport of bacteria to the liver via the gut-liver axis/portal vein. On one hand, enteric-derived NETs exacerbate intestinal barrier damage, while on the other, NETs travel to the liver via the portal vein, where they synergize with NETs produced by liver neutrophils, causing liver injury. As a bridge between gut microbiota and liver inflammation, NETs can directly stimulate liver immune cells by carrying microbial components, driving further hepatic inflammation (58). Thus, the imbalance of gut microbiota, excessive NET activation, and intestinal barrier dysfunction create a vicious cycle (**Figure 2**).

## 5 NETs in NAFLD-HCC

### 5.1 NAFL

NAFL is characterized by hepatic fat accumulation accounting for 5–10% of liver weight and represents the early stage of NAFLD. The primary pathological feature is macrovesicular steatosis involving more than 5% of hepatocytes. Both genetic and environmental factors contribute to NAFLD development, with the gut-liver axis playing a critical role. Disruption of the intestinal barrier and gut microbiota imbalance lead to immune activation, triggering the release of inflammatory cytokines that recruit immune cells, including neutrophils, to the intestine and subsequently to the liver. Neutrophil infiltration has been observed in hepatic lobules during NAFL (111–113).

Obesity and metabolic disorders further exacerbate NAFLD through NET formation. The chronic inflammatory state associated with obesity promotes innate immune activation, enhancing NETosis, which in turn contributes to immune dysregulation, oxidative stress, and metabolic dysfunction. Studies have demonstrated increased spontaneous NET formation in mice on a high-fat diet compared to controls, with obese patients exhibiting elevated plasma NET markers, such as MPO-DNA complexes (113–115). Immunohistochemical analysis has confirmed neutrophil infiltration in the hepatic lobules of STAM mice, and DNase treatment reduced hepatic citH3 expression, suggesting that NET degradation alleviates neutrophil infiltration and hepatic injury. Chronic inflammation, neutrophil activation, NET accumulation, and ROS production form a pathological loop that exacerbates obesity-related liver damage. Increased NET in obese individuals contribute to NAFLD progression by maintaining inflammation, disrupting hepatic energy homeostasis, and promoting hepatocyte lipid accumulation and toxicity.

Metabolic disorders, driven by genetic and lifestyle factors, also modulate NETosis. Patients with type 2 diabetes exhibit increased NETs formation, primarily in response to pro-inflammatory cytokines rather than hyperglycemia itself (116). However, *in vitro* studies suggest hyperglycemia may impair and delay NETosis (117). Hyperlipidemia induces neutrophilia, which correlates with atherosclerosis and related cardiovascular diseases (118, 119). In atherosclerotic mouse models, cholesterol crystals directly induce NETosis or are engulfed by macrophages, triggering cytokine release—particularly interleukin-1β (IL-1β), a key NET inducer. The intracellular mechanisms of neutrophil-cholesterol crystal interaction involve ROS bursts and NE translocation to the nucleus (120). In diabetic and high-fat diet conditions, PAD4 deficiency appears to increase susceptibility to hepatic steatosis, suggesting a potential metabolic role for PAD4. In hyperglycemic patients, glucose may synergize with other stimuli, such as LPS, to enhance NETosis (121). DAMPs released during hepatic ischemia-reperfusion injury promote NET formation via TLR signaling, exacerbating liver damage and inflammation (122).

Regulatory proteins involved in lipid metabolism and inflammation further influence NET formation in NAFLD. Overexpression of Pleckstrin homology-like domain, family A, member 1 (PHLDA1) negatively regulates sterol regulatory element-binding protein 1 (SREBP-1), a key regulator of triglyceride synthesis. Reduced hepatic levels of T cell death-associated gene 51 (TDAG51) correlate with obesity, hepatic steatosis, and insulin resistance (IR), while restoring TDAG51 expression mitigates NAFLD in mice. Machine learning identified activated T cells, macrophages, and neutrophils might play roles in the progression of liver disease (123). TDAG51 also enhances FoxO1 activity in LPS-induced inflammatory responses and promotes NETs release via the TLR4-JNK axis (124). Dysregulated TLR4-mediated inflammation is implicated in various chronic inflammatory diseases, including autoimmune disorders, cancer, and metabolic syndromes (125–127).

Steatosis is linked to cytokine signaling, extracellular matrix interactions, and key inflammatory pathways, including NF-κB, MAPK, and JAK-STAT. NF-κB activation induces NLRP3 inflammasome formation, leading to IL-1β production in response to DAMPs such as cholesterol crystals, ROS, and fatty acids (128–130). These mechanisms activate TLRs, promoting inflammation and fibrosis through NF-κB and MAPK signaling (128–130). Recent studies highlight the JAK-STAT pathway’s role in inflammation, cancer, and neurodegenerative diseases, linking cytokine release and immune regulation to NAFLD pathogenesis (131). The JAK-STAT pathway has also been implicated in NASH progression. Additionally, Wohlmann et al. identified thymic stromal lymphopoietin (TSLP) as an inflammatory mediator in atopic diseases via JAK-STAT signaling (132). Given these findings, pivotal genes such as PHLDA1 and zinc finger protein 36-like 2 (ZFP36L2) may contribute to NAFLD through TLR, MAPK, and JAK-STAT pathways, representing potential therapeutic targets.

**Figure 3:**
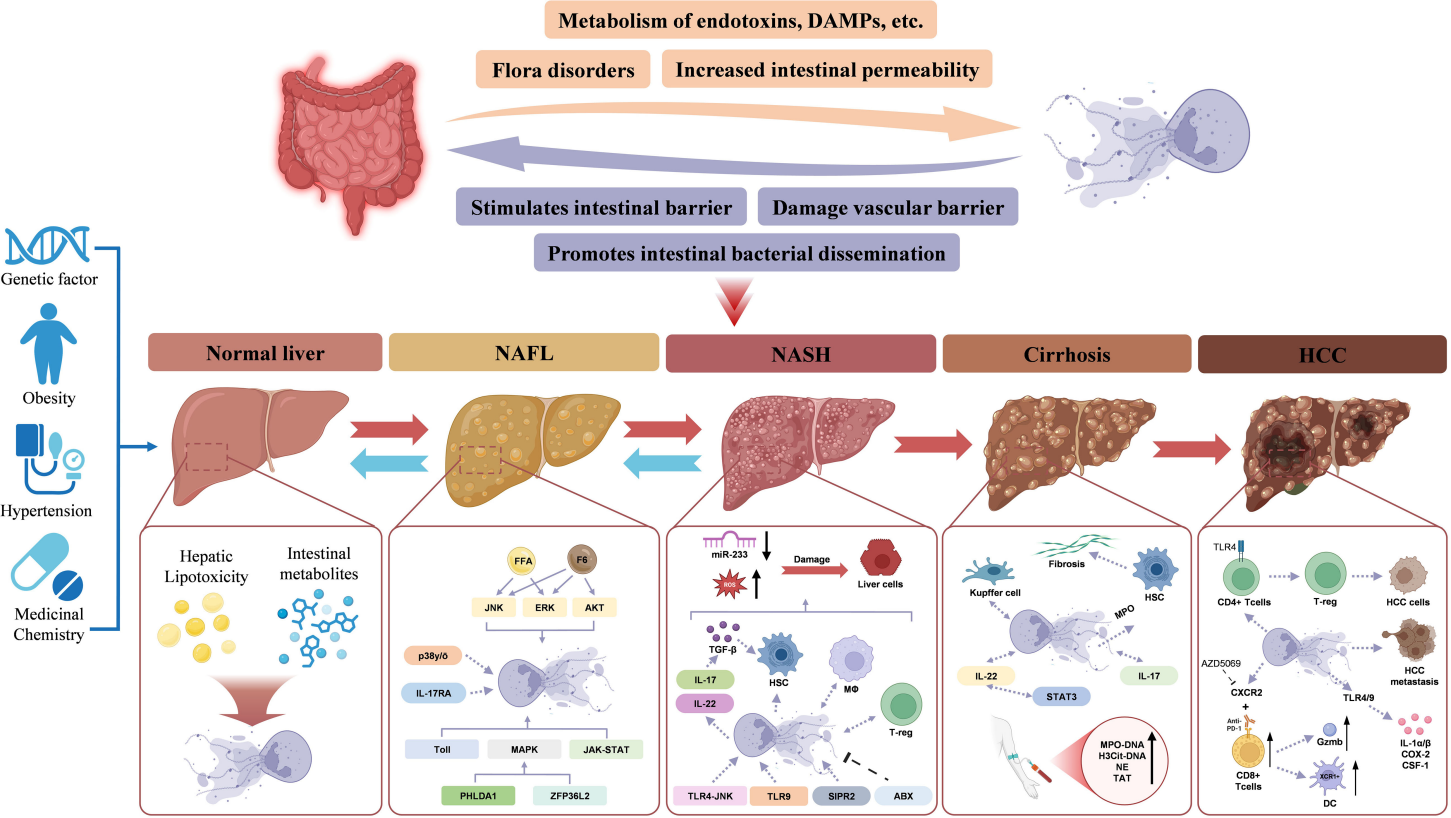
NETs in NAFLD-HCC. The NETs and gut-liver interactions in the progression from a health liver to NAFL, NASH, cirrhosis, and HCC. Normal Liver: Under healthy conditions, the intestinal barrier is intact, preventing endotoxin and metabolite translocation to the liver. Genetic factors, obesity, and hypertension may predispose individuals to liver dysfunction, initiating lipotoxicity and triggering NET formation. NAFLD, Dysbiosis and increased intestinal permeability allow endotoxins, DAMPs, and metabolites to reach the liver via the portal vein. NETs contribute to hepatic inflammation by amplifying lipotoxicity and activating inflammatory pathways like JNK, ERK, and AKT. NASH, With the progression to NASH, NETs exacerbate liver damage by interacting with gut-derived signals. Inflammatory cytokines (e.g., IL-17 and IL-22) activate HSCs, promoting fibrosis. MicroRNA-233 dysregulation further drives hepatocyte injury. Cirrhosis: Kupffer cells and HSCs engage in NET-mediated pathways. Fibrosis progresses due to sustained inflammation and immune activation. HCC: NETs facilitate tumor progression and contribute to HCC cells by activating CD4^+^ Tcells followed by T-reg. NETs, Neutrophil extracellular traps; HCC: Hepatocellular carcinoma; HSCs, Hepatic stellate cells; NAFL, Non-alcoholic fatty liver; NASH, Non-alcoholic steatohepatitis.

Approximately 20% of NAFLD cases progress to NASH, which carries a higher risk of advancing to fibrosis and hepatocellular carcinoma (133). However, with the rising prevalence of metabolic syndrome and its strong association with NAFLD, the global burden of NASH and its complications is increasing at an alarming rate, necessitating urgent attention (**Figure 3**).

### 5.2 NASH

NASH is a more severe form of NAFLD characterized by liver inflammation and hepatocellular injury (steatohepatitis). Unlike NAFL, NASH exhibits both metabolic and inflammatory dysregulation, with pathological hallmarks including hepatocyte ballooning, chronic hepatic inflammation, and progressive fibrosis. It can lead to severe liver complications such as cirrhosis, liver failure, and HCC and may also increase the risk of extrahepatic adverse outcomes. The transition from NAFL to NASH is driven by abnormal lipid metabolism, excessive fat accumulation, and lipotoxicity, with neutrophil infiltration playing a key role in inflammation-induced liver injury. Diets rich in carbohydrates and cholesterol exacerbate neutrophil-driven liver inflammation and are linked to NASH severity.

NETosis is pivotal in the progression from NAFLD to NASH. While blocking NETs does not prevent hepatic steatosis or exsiting free fatty acid (FFA) accumulation caused by factors such as diet, it significantly reduces macrophage infiltration and shifts the inflammatory environment to a less tumor-promoting state. Elevated serum levels of NET markers in NASH patients further highlight the clinical relevance of NETs in NAFLD. Targeting NETs may be a promising strategy for reducing HCC risk in fatty liver disease (134).

NAFLD is associated with increased hepatic FFA levels (135, 136). FFAs are major activators of inflammatory pathways in NAFLD progression, and lipotoxicity-induced lipid accumulation is a key event in hepatic steatosis. Studies have shown that FFAs, such as linoleic and palmitic acid, can stimulate neutrophils to undergo NETosis, while inhibition of fatty acid synthase in human liver tissue prevents hepatic steatosis (137, 138). Experimental models indicate that blocking NET formation does not prevent fat accumulation in the liver, suggesting that NETs are a consequence rather than a cause of hepatic lipid overload. In addition to hepatocyte toxicity, FFAs have been shown to impair CD4^+^ T cells in NASH (139, 140). Several FFAs commonly elevated in NAFLD act as NET stimulators, promoting inflammation, recruiting immune cells such as macrophages and regulatory T cells (Tregs), and driving NASH progression toward HCC (137, 141).

NETosis is an early event in NASH pathogenesis, primarily by shaping the inflammatory microenvironment through monocyte-derived macrophage recruitment. NETs also interact with immune cells to release cytokines, and NET components themselves can directly stimulate hepatocytes, exacerbating NASH. Notably, preoperative serum MPO-DNA levels in NASH patients are significantly elevated compared to individuals with normal liver function. NETs contribute to disease progression by increasing liver macrophage infiltration, as infiltrating macrophages—derived from monocytes recruited in response to inflammation—serve as key effectors in NASH, amplifying cytokine-driven inflammation (142, 143).

Neutrophil infiltration and NET formation occur early in NASH, preceding macrophage accumulation. Blocking NETs significantly alters liver inflammation by reducing monocyte-derived macrophage infiltration, although the precise mechanisms driving neutrophil recruitment into the liver remain unclear (144). NETs themselves may promote neutrophil infiltration, while excessive NET formation and its major component, ROS, contribute to hepatocyte injury. Additionally, reduced miR-223 expression enhances IL-6 production, further exacerbating liver damage and increasing susceptibility to infections in advanced liver disease (145, 146). By establishing a chronic inflammatory liver microenvironment, NETs play a crucial role in NASH progression and HCC development (**Figure 3**).

### 5.3 Fibrosis

Fibrosis represents the next stage in NAFL progression, with epidemiological studies indicating that approximately 20% of NASH patients develop fibrosis annually. As cirrhosis advances, hepatic immune function progressively declines, leading to complications such as portal hypertension, intestinal barrier dysfunction, and bacterial translocation, which can ultimately result in liver failure. During the development of NASH and liver fibrosis, the gut-liver axis, adipose-liver axis, and renin-angiotensin system (RAS) may be dysregulated and impaired (147). Myofibroblasts, pro-fibrogenic mechanisms and cell interactions in progressive NAFLD (9). Hepatic stellate cells (HSCs) and Kupffer cells are the primary mediators of liver fibrosis. MPO has been shown to activate HSCs, thereby promoting fibrotic progression (148). Kupffer cells, the liver-resident macrophages, interact with neutrophils through NETs, exhibiting a dual regulatory effect. Conversely, NETs also influence Kupffer cell function, suggesting a bidirectional interaction. This interplay indicates that NETs may drive liver fibrosis by sustaining a self-amplifying cycle following activation by a priming factor (**Figure 3**).

### 5.4 HCC

HCC represents the terminal stage of NAFLD, with strong epidemiological associations linking NAFLD to primary liver tumors, including NAFLD-related HCC, HCC of other etiologies, and liver metastases from extrahepatic malignancies. HCC accounts for 21–22% of all liver tumors, and the global proportion of HCC cases attributed to NAFLD ranges from 1% to 38%, with the highest risk observed in patients with NAFLD-related cirrhosis (149). In recent years, the increasing prevalence of NASH has contributed to a rapid rise in NAFLD-associated HCC (150). However, because NASH is often undiagnosed or misclassified as “cryptogenic cirrhosis,” and in some cases progresses directly to HCC without an intermediate cirrhotic stage, HCC is frequently detected as an initial clinical manifestation (~35–50%) with rapid disease progression (151). Notably, the stagewise progression of NASH to HCC occurs more frequently than liver cancer arising from other etiologies, suggesting the involvement of systemic or metabolic risk factors unique to NASH (152). Unlike other malignancies, HCC primarily develops in a chronic inflammatory microenvironment (153). Established risk factors for NAFLD-associated HCC include advanced age, male sex, Latino ethnicity, cirrhosis, obesity, and type 2 diabetes, all of which significantly elevate the risk of NASH-related HCC (149). Additionally, emerging evidence implicates gut microbiota dysbiosis and inflammation as key contributors to HCC development in NAFLD (140).

NASH promotes HCC by impairing immune surveillance through the suppression of CD4^+^ and CD8^+^ T cells, increasing intestinal inflammation, and disrupting gut microbiota homeostasis—processes that are pivotal in hepatocarcinogenesis (154). Several recent studies indicate that chronic steatosis induces auto-aggressive CD8^+^CXCR6^+^PD1^+^ T cells that eliminate parenchymal and non-parenchymal cells in an antigen-independent manner promotes chronic liver damage and a pro-tumorigenic environment (155). Under the stimulation of the tumor inflammatory microenvironment (IM), the reprogramming of Treg cells, as members of CD4^+^ T cells, enhances their suppression of immune responses, ultimately promoting tumor immune escape or tumor progression (156). NETs play a central role in shaping the chronic inflammatory liver microenvironment that fosters HCC development. Beyond accelerating NASH progression via the gut-liver axis, NETs are intrinsically involved in HCC pathogenesis. Elevated GM-CSF, a feature of many solid tumors, and LPS released from HCC and intestinal tumors via complement activation contribute to systemic neutrophil activation and NET formation. Increased NETosis, in turn, enhances tumor-associated thrombosis and worsens clinical outcomes. Additionally, NETs act as metastatic scaffolds, facilitating the aggregation of circulating tumor cells in peripheral tissues and “reawakening” dormant cancer cells through NET-associated proteins (157). Experimental studies suggest that DNase treatment exerts antitumor effects by disrupting NETosis.

Metabolic disorders and obesity further contribute to HCC pathogenesis through NET-mediated mechanisms, including DNA damage and oxidative stress. Obesity is a recognized risk factor for HCC in both cirrhotic and non-cirrhotic NAFLD patients, and countries with a rising prevalence of NAFLD-associated HCC typically exhibit higher obesity rates (158). Obesity-driven inflammation promotes liver tumorigenesis by increasing levels of pro-tumor cytokines such as IL-6 and TNF (159). Chronic low-grade inflammation resulting from lipid accumulation induces IL-6 and TNF release, which in turn stimulate NET formation. IL-6 activates the STAT3 pathway, promoting hepatocyte proliferation and malignant transformation (160). However, no definitive evidence links obesity to altered prognosis in NAFLD-related HCC.

Diabetes mellitus is another independent risk factor for HCC, as demonstrated in a large European cohort study involving 136,703 NAFLD patients, where diabetes emerged as the strongest predictor of HCC development (161). While hyperglycemia exacerbates NET-associated inflammation in NASH and HCC, its effects on HCC progression are not exclusive to NAFLD. Additional metabolic alterations in diabetes likely contribute to the elevated HCC risk. Moreover, NAFLD frequently coexists with other liver diseases, such as viral hepatitis and alcoholic fatty liver disease, acting as both a complicating factor and a promoter of occult liver malignancies. Compared to HCC from other etiologies, NAFLD-related HCC is characterized by an older age of onset (median: 73 years), larger tumor size, more aggressive progression, and limited eligibility for curative interventions (162). Epidemiological studies estimate a median overall survival of only 10.7 months for HCC patients (162).

Neutrophils are highly abundant within the HCC microenvironment and display significant heterogeneity. While neutrophils possess antimicrobial, immunoregulatory, and tissue-repair functions, they can also drive tissue damage, immune suppression, and tumor metastasis under specific conditions. In addition to their role in HCC progression via the gut-liver axis, metabolic syndrome, and NASH-HCC, tumor-associated neutrophils (TANs) further contribute by releasing NETs, which promote HCC progression (137). NETs exacerbate the hypercoagulable state associated with cancer by inducing tumor-associated thrombosis, thereby increasing the risk of tumor-related complications, such as organ failure (163, 164). Animal studies have demonstrated that NET depletion slows tumor growth in mice (137).

The liver is also a common site for metastases from colorectal and breast cancer. During gut-liver axis-mediated metastasis, NAFLD facilitates tumor cell dissemination, while the tumor microenvironment reciprocally promotes NAFLD progression. *In vitro* studies indicate that NETs facilitate the invasion and infiltration of metastatic cancer cells into the liver. Clinically, NETs are more frequently observed in colorectal and breast cancer patients with liver metastases than in those without, and elevated NET markers in patient serum serve as potential biomarkers for predicting early liver metastasis in breast cancer (165) (**Figure 3**).

## 6 NETs as a therapeutic target for NAFLD

NETs are closely associated with the conversion of NAFLD to more severe forms of NASH and have been shown to be associated with the development of liver fibrosis, cirrhosis and HCC. Targeted inhibition of the interaction between NETs and the gut-liver axis to suppress the onset and progression of NAFLD is a promising direction for the treatment of NAFLD. Potential therapeutic strategies for targeting the interaction of NETs with the gut-liver axis in NAFLD are summarized below (**Table 2**).

**Table 2:** NETs as a therapeutic target for NAFLD.

	Possible therapeutic drugs	Mechanism of action	Conferences	Published
Reduction of NETs formation	Metformin	Decrease NET DNA release and the numbers of NET mtDNA copies in cultured neutrophils	(166)	2015
	Cyclosporine A	Regulate pentose phosphate pathway	(167)	2023
	Simvastatin	Regulate of the oxLDL/Mac-1 pathway	(168)	2024
	Hesperetin	Regulate of the ROS/autophagy signaling pathway	(169)	2023
	Re-Du-Ning injection (RDN)	Suppress MAPK pathway	(170)	2021
	Neutrophil hijacking nanoplatform (APTS)	Reprogram NETosis to apoptosis in neutrophils via a reactive oxygen species scavenging-mediated citrullinated histone 3 inhibition pathway	(171)	2024
	Resveratrol	Reduce neutrophil activation and free DNA release	(172)	2022
	Inhaled corticosteroids (ICS)	Unclear mechanism	(173)	2020
	Dexamethasone	Inhibit ROS production by leukocytes	(174)	1999
	VitC	Reduction of NF-κB activation to block upregulation of PAD4, ER stress and autophagy signaling genes	(175)	2013
Promote degradation of NETs	Dnase I	Recognise and cleave DNA strands in NETs, thereby disrupting the structural integrity of NETs	(137)	2018
	Alpha-1-antitripsin (AAT)	Change the shape and adherence of non-COVID-19-related NETs;augments neutrophil superoxide production, inhibiting the activity of neutrophil elastase	(176)	2021
Regulating gut microbiota	β-arbutin	Inhibiting the overrecruitment of neutrophils and the overrelease of NETs by inhibiting the ErK signaling pathway	(177)	2024
	Butyrate	Inhibit ROS production by leukocytes	(178)	2021
	Antibiotics	Microbiota depletion	(179)	2015
	Lactobacillus rhamnosus	Inhibite ROS production and phagocytosis by neutrophils	(180)	2014
Multi target combination therapy	Aspirin/hydroxychloroquine in combination with DNase 1	Block COX2 and upstream TLR4/9 activation complementary to DNase I	(181)	2020

### 6.1 Reduction of NET formation

In recent years, several therapeutic strategies have emerged that target and inhibit the formation of NETs. In a clinical trial, metformin was found to reduce PMA-induced NETs formation (166). Tradition Chinese medicine preparations such as Re-Du-Ning (RDN) injection have also been shown to meliorate LPS-induced ALI through suppressing MAPK pathway to inhibit the formation of NETs (170). The study found that in septic mice, Hesperetin treatment reduced PMA-induced ROS production and NET formation, thereby attenuating sepsis-induced intestinal barrier damage (169). Simvastatin reduced hyperlipidemia-induced hepatic IRI by inhibiting the formation of NETs through the regulation of the oxLDL/Mac-1 pathway (168). Cyclosporine A (CsA) may reduce the severity of colitis by reducing the formation of NETs *in vivo*. *In vitro*, CsA reduces the release of ROS-dependent NETs by directly decreasing G6PD activity through activation of p53 protein, which downregulates PPP and cellular ROS levels (167). Antioxidant drugs, such as resveratrol, have been shown to be effective in reducing NETs produced by neutrophils in severely COVID-19-infected individuals by decreasing the neutrophil activation state and free DNA release (172). Inhaled corticosteroids (ICS) have also been shown to significantly reduce the formation of NETs, and in asthmatics, plasma NET levels were significantly lower in patients treated daily with ICS than in patients who used no or little ICS, but the mechanism by which they inhibit NETs formation is unknown (173). In another study, a significant reduction in reactive oxygen species production was observed in neutrophils after intravenous dexamethasone administration in human healthy subjects (174). And it’s well known that NETosis depends on the production of reactive oxygen species (182). Some studies have shown that anti-inflammatory drugs also have the ability to lower NETs (181). Significant improvement in fibrosis grading was also observed in the rat model of liver fibrosis in the aspirin and enoxaparin treatment groups (183).

Vitamin C (VitC), also known as ascorbic acid, is a water-soluble vitamin that is essential for human health. Studies have shown that in ascorbic acid deficiency, upregulation of hypoxia-inducible factor-1α (HIF-1α) blocks neutrophil apoptosis under normoxic conditions (184). Specifically, VitC may block the upregulation of PAD4, ER stress, and autophagy signaling genes by reducing NF-κB activation, thereby attenuating NETosis; in this way, VitC also significantly attenuated PMA-induced NETosis in polymorphonuclear (PMN) of healthy human volunteers (175). The antioxidant properties of VitC help to reduce oxidative stress, thereby reducing the formation of NETs (185). Interestingly, studies reporting an efficient neutrophil hijacking nanoplatform (referred to as APTS) for targeted A151 (a telomerase repeat sequence) delivery to microglia to dramatically reduce the formation of NETs by 2.2-fold via reprogramming NETosis to apoptosis in neutrophils via a reactive oxygen species scavenging-mediated citrullinated histone 3 inhibition pathway (171).

### 6.2 Promoting degradation of NETs

A preclinical study demonstrates the potential of recombinant human DNase I to treat cancer-related thrombosis (186). In a mouse model of necrotizing fasciitis, Group A Streptococcus (GAS) expressing DNase Sda1 has been identified as a contributor to bacterial virulence. Sda1 efficiently catabolizes NETs both *in vitro* and *in vivo* (54). DNase has shown therapeutic potential in animal models of NASH-HCC (137). However, DNase I for NETs alone has limitations. Blood concentrations of a given DNase I were found to be less stable (187). In addition, DNase I disrupts the NET structure but does not completely degrade the protein components of the NETs, suggesting that it is less effective in eliminating the inflammatory response triggered by NETs (58).

The other naturally occurring molecule, reducing pathological NET activity is alpha-1-antitripsin (AAT), a neutrophil elastase inhibitor, capable of change the shape and adherence of non-COVID-19-related NETs (176). AAT binds extracellular IL-8, reducing the neutrophils’ influx to the inflammatory site and augments neutrophil superoxide production, inhibiting the activity of neutrophil elastase (176).

### 6.3 Regulating gut microbiota

Numerous studies have shown that gut microbiota imbalance disrupts gut homeostasis and increases the risk of advanced NAFLD, and that this imbalance triggers hepatic inflammation and injury via the gut-liver axis, which affects bile acid metabolism and fat accumulation, and exacerbates liver fibrosis (188–193). Activation of NETs by microbiota has been reported (194). NETs were observed to be activated in a rat model of LPS-induced sepsis, and disruption of these NETs was found to attenuate intestinal damage (108). Microbiota-derived metabolite butyrate was found to inhibit neutrophil migration and NET formation in patients with Inflammatory Bowel Disease (IBD) (178). β-arbutin, a glycoside extracted from the Arctostaphylos uva-ursi leaves has also been found to contribute to the maintenance of intestinal homeostasis by inhibiting the formation of NETs, maintaining the integrity of the mucosal barrier, and shaping the composition of the intestinal flora (177).

One study focused on the effect of gut microbiota on NETs. It was found that the use of a combination of antibiotics that included ampicillin, streptomycin, metronidazole, and vancomycin reduced the number of microbes in the gut. This reduction correlated with a decrease in the formation of NETs. This means that by reducing certain types of bacteria, there may be an indirect reduction in the production of NETs in the body (179). Certain probiotics such as Lactobacillus rhamnosus strain GG have also been found to inhibit PMA and S. aureus-induced NET formation (180). These findings suggest that gut microbiota-targeted therapies hold promise as potential interventions to limit the formation of NETs during NAFLD (195, 196).

### 6.4 Multi target combination therapy

NETs promote HCC metastasis by activating the tumor inflammatory response, and some researchers have used two anti-inflammatory drugs, aspirin and hydroxychloroquine, to block the activation of cyclooxygenase 2 (COX2) and upstream TLR4/9 in combination with DNase I, and have demonstrated their promising effects in inhibiting HCC metastasis from multiple perspectives (181). The ability of these combination therapies to block or break down NETs and eliminate the metastatic potential of HCC cells trapped by unresolved NETs demonstrates a new use for old anti-inflammatory drugs. More strategies for combining with DNase I to combat metastasis need to be developed in the future. It is difficult to conclude that one compound works better than another in the treatment of targeted NETs, so more research is needed. Management of NETs may require the use of combination therapies that incorporate conventional treatments (e.g., fluid therapy, antibiotics, antivirals, and NET-targeting drugs) (111). Another avenue is integrating NET-targeting agents with antifibrotic compounds such as obeticholic acid or selonsertib. Since NETs can promote hepatic stellate cell activation and fibrogenesis, inhibiting NET formation while simultaneously targeting fibrogenic signaling may produce synergistic effects (197, 198).

## 7 Outlook

With the deepening understanding of the pathogenesis of NAFLD, it is increasingly recognized that gut microbiota dysbiosis and gut-liver axis dysfunction are among the important factors in the development of NAFLD (199, 200). Therefore, future therapeutic strategies should not only focus on the inflammatory response of the liver itself, but also consider how to intervene in the disease process by regulating the intestinal microecological balance. This provides a wide scope for the development of new therapeutic approaches. The following aspects deserve special attention:

### 7.1 Applications of nanomaterials

Nanoparticle delivery systems: Nanotechnology provides a new platform for drug delivery systems that can improve drug bioavailability and reduce side effects. The design of targeted nanoparticles for carrying enzymes or other active ingredients that degrading NETs promises to be an innovative therapeutic tool. For example, nanoparticles loaded with DNase I have shown significant therapeutic effects in animal models (201). Future research could further optimize the design of nanocarriers to enable more precise delivery of drugs to the liver or gut, and thus more effective against NETs-mediated inflammation and injury.

Multifunctional nanomaterials: In addition to single-function nanoparticles, multifunctional nanomaterials can be developed to combine multiple therapeutic mechanisms, such as carrying both anti-inflammatory drugs and NETs-degrading enzymes, to achieve synergistic effects.

### 7.2 Traditional Chinese medicine and natural products

Traditional Chinese medicine (TCM) has accumulated a wealth of experience in regulating immune responses and ameliorating chronic diseases. Some TCM such as Xuanfei Baidu Decoction (XFBD) have shown potential to regulate NETs formation via CXCL2/CXCR2 axis. Continued exploration of the active ingredients of TCM and their mechanisms of action may reveal more natural products that can be used for the treatment of NAFLD. For example, herbal components such as baicalein and tanshinone have been shown to have anti-inflammatory and antioxidant effects (202, 203).

### 7.3 Exosome research

Regulatory role of exosomes: exosomes, as important mediators of intercellular communication, play an important role in regulating immune responses and tissue repair processes. Studies have shown that certain types of exosomes can influence the production and clearance of NETs (204). Therefore, understanding how exosomes from different sources affect the function of NETs and how they can be used to optimize therapeutic regimens will be a key area for future research. For example, exosomes from stem cells have been shown to reduce liver inflammation by modulating immune cell function (205, 206).

Exosomes as therapeutic carriers: Exosomes can be used not only as therapeutic targets but also as drug delivery carriers. Future research could explore the use of exosomes to deliver specific drugs or enzymes to achieve more precise therapeutic effects. For example, by modifying the surface of exosomes so that they can be specifically targeted to the liver or intestines, thereby increasing the local concentration and efficacy of the drug (207, 208).

### 7.4 Microbiomics and personalized therapy

With the deepening of microbiomics research, there is increasing evidence of the key role of gut flora in the pathogenesis of NAFLD. Analysis of patients’ gut flora by high-throughput sequencing technology can help identify specific microbial markers and provide a basis for achieving precision medicine based on individualized characteristics (209). Future studies could further explore how to improve the metabolic status and liver health of NAFLD patients by modulating the gut microecology (e.g., using prebiotics, probiotics, or fecal bacteria transplantation).

Although the above emerging areas show great potential, it is difficult for any single approach to comprehensively address the complexities of NAFLD. Therefore, lifestyle modification, weight control, and the use of known effective medications should not be overlooked while exploring new therapies. For example, weight reduction through exercise and dietary changes, and the use of medications such as metformin to enhance insulin sensitivity are all approaches that have been shown to be effective in alleviating NAFLD symptoms (210–212). Combining these foundational measures with new strategies for targeting NETs is expected to have a synergistic effect and significantly improve the overall health of patients.

## 8 Conclusion

In summary, NETs play an important role in the genesis and development of NAFLD. The gut-liver axis plays an important role in the initiation of NAFL, which is mainly caused by the disruption of the intestinal barrier and the imbalance of the gut microbiota, and NETs continue to accelerate the process of hepatic fibrosis through the self-circulation of NETs activated by the stimulation of the gut-liver axis, gene induction, lipotoxicity accumulation, and the initiation of metabolic diseases. From healthy liver to NAFLD, NASH, liver fibrosis and even HCC, the formation and release of NETs is one of the key factors connecting these pathological stages. NETs are not only directly involved in hepatic inflammatory response and tissue injury, but also interact with gut microbiota through the gut-liver axis, which further promotes the disease process.

Studies have shown that an imbalance in the gut microbiota is able to exacerbate the process of liver inflammation and fibrosis by disrupting the intestinal barrier function and increasing the transfer of bacterial products to the liver (3, 213). In addition, specific metabolites in the intestinal microenvironment may regulate the production and degradation of NETs and influence the progression of NAFLD (213). Therefore, when treating NAFLD, in addition to focusing on the inflammatory state of the liver itself, the role of the gut-liver axis and gut microbiota needs to be considered comprehensively for a more comprehensive and effective management strategy.

Targeting NETs as therapeutic targets, approaches to reduce the formation of NETs, promote their degradation, modulate the gut microbiota composition, and multi-targeted combination therapies have demonstrated potential applications. In particular, modulating the gut microbiota to indirectly alleviate liver inflammation by improving gut health provides a new therapeutic perspective for NAFLD patients. Future studies should continue to explore the specific mechanisms of NETs in different stages of NAFLD-HCC and develop more new therapeutic approaches based on gut-liver axis modulation, with the aim of improving the prognosis of patients and reducing the disease burden.
